# Textural Classification of Commercial Foodstuffs for Dysphagia Using Back-Extrusion Test

**DOI:** 10.3390/foods14213741

**Published:** 2025-10-31

**Authors:** María Teresa Murillo-Arbizu, Leyre Urtasun del Castillo, Sandra González-Casado, Juan Jesús Marín-Méndez, Francisco C. Ibañez, María José Beriain

**Affiliations:** 1 Institute for Sustainability & Food Chain Innovation (IS-FOOD), Public University of Navarra (UPNA), Jerónimo de Ayanz Building, Arrosadia Campus, 31006 Pamplona, Navarre, Spain; mariateresa.murillo@unavarra.es (M.T.M.-A.); mjberiain@unavarra.es (M.J.B.); 2 National Centre for Food Technology and Safety (CNTA), Crta-Na-134-Km 53, 31570 San Adrián, Navarre, Spain; lurtasun@cnta.es (L.U.d.C.); sgonzalez@cnta.es (S.G.-C.); jmarin@cnta.es (J.J.M.-M.)

**Keywords:** textural property, instrumental texture, texture-modified food, swallowing disorder, IDDSI framework

## Abstract

Oropharyngeal dysphagia (OD) management requires texture-modified foods (TMFs). The International Dysphagia Diet Standardization Initiative (IDDSI) framework classifies TMFs from drinks (levels 0–2) to purées and soft-solid foods (levels 3–4). However, current instrumental methods for analyzing commercial OD-oriented TMFs often fail to provide reliable classifications, limiting their clinical and industrial applicability. This study aimed to evaluate the effectiveness and reliability of the Back-Extrusion Test (BET) in classifying commercial OD-oriented TMFs according to the IDDSI framework. Fifty-four commercial TMFs were analyzed using BET1 method (firmness and adhesiveness), and BET2 method (firmness, consistency, cohesiveness, and cohesion work). A progressive increase in firmness and consistency was detected as IDDSI level rose, with significant differences between levels. The classification accuracy for IDDSI levels, as determined by discriminant analysis, was 66.1% (BET1) and 76.8% (BET2), although both methods showed reduced performance, particularly for level 4 foods. Cluster analysis revealed three groups by means of BET1 and BET2, identifying levels of foods with low, intermediate, and high textural complexity. This finding suggests that a simplified classification framework could improve objectivity and reliability in assessing OD-oriented TMFs. Furthermore, integrating additional instrumental techniques may improve the accuracy classification of commercial foods where BET methods fail.

## 1. Introduction

Oropharyngeal dysphagia (OD) is a medical condition characterized by difficulty in swallowing, which may arise from an inability to adequately process food in the mouth or from dysfunction in the swallowing mechanism in the upper gastrointestinal tract. Contributing factors can include age, impaired mobility, structural abnormalities, and neurological disorders among others. Uncontrolled OD can result in clinical issues such as dehydration and malnutrition, as well as more serious complications like aspiration pneumonia, choking, and, in severe cases, premature death [1]. As a result, it is critical to develop safe and effective strategies for managing OD, and texture-modified foods (TMFs) play a central role in this approach. Modifications to fluid consistency and food texture are commonly used strategies to aid safe swallowing in people with OD. “Thickened fluids,” such as thickened beverages, and “texture-modified foods,” like purées or minced foods, are designed to alter the mechanical properties of drinks and foods [2].

In response to the increasing need for standardized classifications of TMFs, the International Dysphagia Diet Standardization Initiative (IDDSI) developed a comprehensive framework to describe both drinks and foods. The framework [3] defines eight levels, categorizing beverage thickness (levels 0–4) and food textures (levels 3–7). It provides simple and effective tools, such as the syringe flow test and the fork drip test, which are highly valuable for caregivers, clinicians, and food industry applications. However, these methods would benefit from a robust scientific rationale. Establishing correlations with objective rheological measurements would not diminish their practicality; instead, it could enhance reliability, efficacy, and potential applications in labeling foods intended for people with OD. The high reproducibility of objective instrumental methods is especially valuable for quality assurance in the food industry [4]. Previous studies have highlighted divergences where thickened liquids labeled by manufacturers as “nectar-like” were classified as thicker consistencies when assessed with IDDSI methods [5]. Furthermore, the limited availability of standardized quantitative data in scientific literature constrains researchers’ ability to systematically examine the effects of food texture on swallowing function. Reliance solely on qualitative assessments also makes it difficult to detect subtle variations in texture and consistency within the same IDDSI level [6]. Therefore, the establishment of instrumental methods capable of providing quantitative data on the textural properties of foods could serve as a valuable tool for the development of TMFs.

The textural characterization of foods developed for people with OD has been investigated using a variety of instrumental and sensory methodologies [4]. However, most research efforts have focused on the instrumental characterization of laboratory-prepared products [6,7], while studies involving commercial products intended for oral dietary feeding remain scarce [8], despite the fact that commercial foods often exhibit more complex structures than those developed in the laboratory. For instance, Poursani & Razavi [9] evaluated the friction, consistency, yield stress, and strain-stiffening of fluids based on xanthan gum and cress seed gum by combining tribological and rheological analyses. However, their findings were limited to lab-prepared fluids and therefore did not capture the behavior of commercial foodstuffs. This suggests that tribology can be a useful tool for investigating the friction and lubrication properties of foods during oral processing. Nevertheless, its application to commercial TMFs remains limited, as the required equipment is expensive and the analyses are complex to perform and interpret. The study performed by Ettinger et al. [10] provided a comprehensive evaluation of ten commercial pureed foods intended for people with OD. Their results demonstrated significant variability in texture, nutritional composition, and sensory attributes among commercially available samples, underscoring the absence of standardization in textural characteristics from an instrumental standpoint, as well as inconsistencies in the methodologies used for their assessment. Previous studies have shown that the Back-Extrusion Test (BET) is a fast, and objective method for measuring textural properties of foods. However, while BET is well-established for certain applications, its reliability and reproducibility for classifying commercial TMFs has not yet been explored [11]. Its use could enable the classification of these foods according to their textural properties.

Given this context, a broader and more comprehensive evaluation is required to determine the effectiveness of instrumental techniques in classifying commercial food samples based on their textural attributes. Methods like the BET provide valuable quantitative data; however, their application specifically to commercial OD-oriented products has yet to be thoroughly investigated. The hypothesis of this study was that BET is a reliable and reproducible method, of interest to the food industry, as it facilitates the classification of commercial OD oriented TMFs based on their mechanical properties and composition. The specific objectives of the study were (i) to evaluate the potential of the BET in classifying commercial OD-oriented TMFs as per the IDDSI framework; (ii) to compare the performance of two BET methods in classifying OD-oriented TMFs; and (iii) to assess whether the existing IDDSI levels can be complemented with a textural classification based on the best BET method.

## 2. Materials and Methods

### 2.1. Selection and Preparation of Food Products

A thorough search was carried out in supermarkets, pharmacies, and online retailers from November 2022 to May 2023 to identify products marketed toward people with OD or deemed suitable for consumption by this population. A total of 54 commercial foodstuffs from different brands were selected. Of these, 35 were “ready-to-eat” items packaged in glass or plastic containers, while the remaining products were powder-based requiring reconstitution in water or milk, according to the manufacturer’s instructions. The research team classified the commercial products into seven categories based on their appearance: jellies (*n* = 7), beverages (*n* = 21), purées (*n* = 10), cream (*n* = 6), custard (*n* = 1), yogurt (*n* = 1), and minced dish (*n* = 8). All products were stored at room temperature or under refrigeration in accordance with the manufactures’ recommendations and were prepared at the time of analysis following supplier guidelines.

The jellies were sweet-tasting preparations primarily composed of water and thickening agents. The carbohydrate-based thickening agents were the main contributors to the nutritional profile, resulting in a composition predominantly made up of carbohydrates, with negligible amounts of other macronutrients. One jelly sample, however, was specifically formulated as protein-enriched, providing 6 g of protein per 100 g product. Among the beverages, the category included milkshakes, thickened drinks with texturizing agents, and nutrient-dense formulations. Notably, 10 beverages had protein concentrations exceeding 6 g/100 g, with one reaching 23 g/100 g. As for purées, most (*n* = 8) were fruit-based. One formulation was lentil-based, and one was cereal-based. Of the six creams analyzed, five were vegetable-based, and one was a high-protein mixture with a protein content of 9 g/100 g. The minced dishes were formulated with the following primary ingredients: animal meat (*n* = 5), vegetables (*n* = 1), fish (*n* = 2), and egg (*n* = 1).

Following sample collection, a dataset was created using a spreadsheet (Microsoft© Excel^®^ 2019, Microsoft Corp., Redmond, WA, USA), categorizing the products into these seven defined groups while recording their nutritional composition and the occurrence of food additives. Those identified included modified thickening, gelling, and texturizing agents such as corn starch, locust bean gum, pectin, xanthan gum, carrageenan, guar gum, and gelatin; modified starches such as acetylated distarch phosphate (E1414) and hydroxypropyl distarch phosphate (E1442); emulsifiers and stabilizers including mono- and diacylglycerides of fatty acids (E471) and soy lecithin; salts and mineral additives such as potassium chloride and disodium diphosphate (E450i); and bulking agents like maltodextrin.

### 2.2. Back Extrusion Test

Two different back extrusion test (BET) procedures were employed. Method 1 (BET1), performed at the Public University of Navarra, was conducted following the method proposed by Ibañez et al. [8], with some modifications. Analyses were carried out using a TA.XT Plus Texture Analyzer (Stable Micro Systems, Surrey, UK), equipped with a 5 kg load cell and an aluminum back-extrusion cylindrical probe (35 mm diameter; model P/35). Prior to testing, the apparatus was calibrated for force using a 2 kg weight, followed by height calibration to ensure accurate measurement parameters. Samples were placed in methacrylate cells (50 mm inner diameter and 60 mm height; model A/BE) and filled to a height of 50 mm. Test conditions were as follows: trigger force, 0.049 N; test distance, 30 mm (corresponding to a 60% strain level); pre-test speed, 10 mm/s; test speed, 5 mm/s; post-test speed, 10 mm/s. Based on previous research on the rheological and textural properties of OD-oriented foods [12,13,14], three temperatures were selected to emulate conditions analogous to food consumption: 5 °C (refrigerated products), 20 °C (room-temperature products), and 40 °C (warmed products). Measurements were performed in triplicate to ensure the reliability and reproducibility of the results. Data obtained from BET1 included maximum force and minimum force, recorded from the force-time profile and expressed in Newtons (N). Data capture and analysis were carried out using Exponent Lite v.6.1 software (Stable Micro Systems Ltd., Surrey, UK). The maximum positive force observed during the compression cycle was associated with sample firmness, whereas adhesiveness was defined as the minimum negative force recorded during the upstroke phase of the test [15].

Method 2 (BET2), conducted at the National Center for Food Technology and Safety (CNTA), was carried out using a TA-XT2 Plus C Texture Analyzer (Stable Micro Systems, Texture Technologies Corporation, Scarsdale, NY, USA), which was also fitted with a 5 kg load cell. The BET followed the method described by Syahariza & Yong [16], with some modifications. Fifty mL of each sample were placed into a 60 mm diameter methacrylate container. The sample was extruded with a 40 mm diameter methacrylate back-extrusion disk (A/BE-D40) for a gap of 20 mm under the following settings: pre-test speed, 1 mm/s; test speed, 1 mm/s; post-test speed, 10 mm/s. Before testing, the apparatus was calibrated for force using a 2 kg weight, followed by height calibration to ensure accurate measurement parameters. The parameters measured included the maximum positive peak force representing firmness (N), and the positive area of the force-time curve, indicating consistency (N × s), both obtained during the compression phase. Cohesiveness was defined as the maximum negative peak force, and the work of cohesion as the negative area under the curve, both recorded during the upstroke phase, when the probe was withdrawn from the sample (N and N × s, respectively). All data were recorded using Exponent Connect Software Version 8.1.8.0 (Stable Micro Systems Ltd., Surrey, UK). Each product was tested in three replicates at the designated serving temperature (either 40 °C, 20 °C or 5 °C).

### 2.3. IDDSI Flow Test

The IDDSI flow test was conducted by CNTA using a single-use 10 mL syringe (ISO 7886-1:2017) [17], with a measurement length of 61.5 mm from the zero line to the 10 mL mark for each test. The procedure adhered to the guidelines outlined by IDDSI framework [18]. Although the IDDSI framework does not provide specifications regarding test temperature, the procedure was standardized by conducting the flow test at the same serving temperatures applied in the texture analyses (5 °C, 20 °C or 40 °C, according to the particular product) to ensure comparison of results. Specifically, 10 mL of each sample were taken with a syringe once the serving temperature was reached, allowed to flow freely for 10 s, and the residual volume retained in the syringe was subsequently measured. Classification according to the IDDSI level (hereafter referred to as ‘level’) was based on the remaining volume as follows: level 0, less than 1 mL remaining; level 1, 1–4 mL remaining; level 2, 4–8 mL remaining; level 3, 8–10 mL remaining; and level 4, no flow, with the syringe remaining full. Each product was tested in triplicate, and the average was calculated. In cases where the measurements differed by more than 1 mL, an additional confirmatory measurement was performed, and the three closest values were averaged [19]. For reliability, all flow tests were conducted by a single evaluator.

### 2.4. Statistical Analysis

All statistical analyses were performed using SPSS Statistics for Windows, version 28 (IBM Corp., New York, NY, USA). Data (*n* = 3) obtained from the two BET methods were evaluated using one-way ANOVA, and variance homogeneity was verified by Levene’s test. Differences among multiple means were identified using Dunnett’s C test (*p* ≤ 0.05), after confirming unequal variances.

For multivariate analyses, the data from each sample were averaged. A quadratic discriminant analysis (QDA) was conducted using instrumental texture data to assess whether each BET method could classify the foods according to their original IDDSI level. The Mahalanobis distance was calculated to verify multivariate normality for each BET method, and the χ^2^ distribution was used for critical values, with freedom degrees equal to 2 for BET1 and 3 for BET2. Wilks’ Lambda test for equality of means and Box’s M test for homogeneity of variance-covariance matrices (*p* ≤ 0.05) were also considered.

Hierarchical cluster analysis (HCA) was conducted to explore natural groupings of commercial products based on similarities in their textural properties. Specifically, clustering was performed using, as independent variables, the parameters obtained from BET1 (firmness 1 and adhesiveness) and BET2 (firmness 2, consistency, cohesiveness, and cohesion work). Ward’s linkage method combined with squared Euclidean distance as the dissimilarity measure was applied, and dendrograms were obtained. Each individual sample was treated as a case. Food classifications into a minimum of three clusters and a maximum of five clusters (corresponding to the IDDSI levels considered) were analyzed.

## 3. Results and Discussion

**Figure 1:**
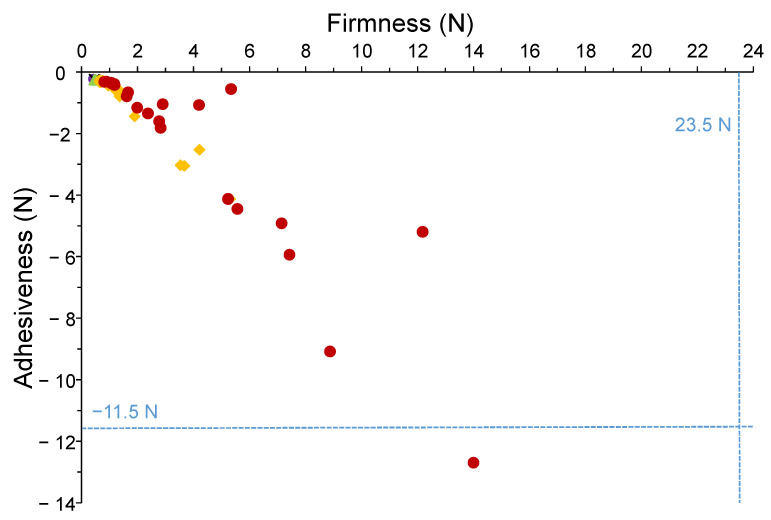
Scatter plot for firmness (mean) and adhesiveness (mean) of the TMF samples analyzed by BET1 method (■ IDDSI level 0; ▼ IDDSI level 1; ▲ IDDSI level 2; ♦ IDDSI level 3; ● IDDSI level 4). The more negative the values on the *Y*-axis, the higher the adhesiveness. The dotted blue line (−11.5 N) indicates the maximum value of adhesiveness for OD-oriented TMFs suitable for people with cerebral palsy (Merino et al. [15]).

Table S1 details the sample type and description, analysis temperatures, IDDSI levels, and outputs for BET1 and BET2 parameters assessed in this study. The samples displayed textural properties within ranges considered safe for people with OD associated with cerebral palsy. For BET1 method, the maximum values recorded (firmness: 14 N; adhesiveness: −12.70 N) corresponded to a minced chicken and carrot preparation yet remained below the critical thresholds of 23.5 N and −11.5 N established by Ibañez et al. [8] and Merino et al. [15] (Figure 1). These findings indicate that the tested foods are appropriate for consumption by people with cerebral palsy-OD. Compared to previous research, firmness values of 0.25–1.01 N observed for rice porridges prepared with various thickeners at different concentrations [16] were consistent with those obtained for the cream-like textures evaluated in the present study. Similarly, Ettinger et al. reported firmness values for commercially prepared pureed carrot (0.5–3.5 N) and turkey (1.2–9.7 N), which are comparable to the range found in the present study (0.77–8.87 N), despite differences in the BET methodology [10].

Table 1 summarizes the ranges of textural parameters for commercial food samples classified according to their IDDSI levels.

Firmness 1 and 2, consistency, and cohesiveness systematically increased across IDDSI levels 0–4, from minimal resistance in level 0 to the highest in level 4 maintaining structural integrity for safe swallowing. Level 1 (slightly thick) and level 2 (mildly thick) foods showed incremental increases in firmness, consistency, and cohesiveness, supporting bolus integrity. For these attributes—adhesiveness, cohesiveness, and cohesion work—more negative values corresponded to higher stickiness, greater cohesiveness, or more energy required to deform the sample, reflecting stronger structural and adhesive properties. Level 3 (moderately thick/liquidized) bridged drinkable and spoonable textures. Cohesiveness varied across matrices, with levels 3 and 4 showing greater variability, reflecting influences beyond particle size, such as the concentration of hydrocolloids, water-binding capacity, and overall internal structure, which collectively enhance the cohesive properties of higher-level TMFs [6]. Adhesiveness generally increased with IDDSI levels, while variability and overlap at certain levels reflect the combined effects of sample properties and measurement methodology [20].

Statistical analysis of BET1 and BET2 measurements confirmed a clear, progressive increase in textural parameters with ascending IDDSI levels (Table 2). Firmness (BET1 and BET2), consistency (BET2), adhesiveness (BET1), and cohesiveness-related parameters (BET2) all differed significantly between levels (*p* < 0.05), reflecting the expected increase in structural resistance and internal binding from liquids to soft solids. Variability was higher at levels 3 and 4, particularly for BET1 firmness (SD = 3.75 N) and BET2 consistency (SD = 15.49 N × s), indicating substantial differences among commercial products within the same category. These trends are closely linked to the compositional and structural properties of the foods. Beverages from low-IDDSI levels (0–1) showed low firmness and minimal adhesiveness or cohesion work due to their high water content and low polysaccharide or protein gel content [6]. In contrast, gelled water products and protein-enriched jellies (level 4) exhibited high firmness and cohesion work, reflecting stable three-dimensional networks formed by hydrocolloids and protein interactions [21]. Fruit and vegetable purées (levels 3–4) displayed intermediate texture values, influenced by fibers, pectin, and starch, which enhance viscosity and structural integrity [22], while minced dishes with protein and complex carbohydrate matrices showed the highest cohesiveness and consistency, resulting from combined effects of muscle fibers, starch, and emulsified fats reinforcing the internal structure [23]. While these instrumental findings support the IDDSI classification by revealing a general trend of increasing textural resistance with higher levels, they also highlight the method’s limitations. The IDDSI framework, based on qualitative empirical tests such as the syringe flow test, assigns samples to discrete categories primarily by assessing viscosity and gravity-driven flow behavior using fixed thresholds and subjective observation. This approach can mask subtle textural variations between products within the same level. In contrast, BET1 and BET2 provide quantitative data on mechanical properties such as firmness and cohesiveness under controlled shear or compression, enabling detection of nuanced differences arising from ingredient composition, processing, and microstructure. As a result, some overlap in BET measurements across adjacent IDDSI levels was observed, underscoring that, while IDDSI serves as a practical tool for clinical classification, instrumental methods reveal a more continuous spectrum of textural and rheological properties within and between levels.

The performance of the two BET methods for classifying samples according to their respective IDDSI levels was evaluated using quadratic discriminant analysis. For BET1, the first canonical function (F1) explained 98.4% of the sample variance and was associated with firmness, while the second canonical function (F2) accounted for 1.6% of the variance and was related to adhesiveness (Figure S1). In the case of BET2, F1 explained 92.2% of the variance and was associated with firmness (negatively) and consistency, while F2 accounted for 7.8% of the variance and was related to cohesiveness and cohesion work (negatively) (Figure S2). Using BET1 method, 66.1% of the samples were correctly classified into their IDDSI level (Table 3), while BET2 method correctly classified 76.8% (Table 4). The two methods exhibited high classification success for levels 0, 1 and 3. However, TMF samples from levels 2 and 4 were less accurately classified. Specifically, half of the level 2 samples were misclassified as level 3. It should be noted that only two samples out of all the samples tested were level 2, displaying the reduced availability of commercial foodstuffs at this level and thus limiting the representativity of results for level 2. Regarding level 4 samples, 63.6% (BET1) and 45.5% (BET2) were classified level 3.

Misclassification of food samples between IDDSI levels and BET results can be attributed primarily to their mechanical properties, which are determined by composition and microstructure. For instance, foods classified as level 4 according to the IDDSI framework, such as fruit and vegetable purées, contain high levels of water and/or sugars, low protein or fat content, and thickening, gelling or texturizing agents. These factors influence key mechanical properties such as cohesiveness, firmness, and resistance to deformation of food. While the food additives allow the products to meet the syringe flow test criteria, their relative low intrinsic cohesiveness or firmness, attributable to reduced protein content, can lead to underestimation of textural level as determined by BET measurements [6,10]. Conversely, protein-enriched formulations including meat purées, protein jellies or plain yogurt, develop firmer, elastic gel networks due to the presence of myosin/actin, collagen or caseins, respectively. Although these structures may appear to be cohesive during a steady, low-stress test, they can become fragmented under the higher shear and compression conditions of BET, at times resembling the behavior of level 3 structures [2]. In addition, products formulated with hydrocolloids or modified starches may exhibit gummy or plastic-like textures that respond inaccurately in IDDSI and BET assessments [9,13]. The matrix heterogeneity, including the presence of dietary fiber or protein fragments, may explain the mismatches. These components may partially obstruct flow in the syringe test; however, they provide less resistance to extrusion in BET, which is associated with reduced cohesiveness.

Overall, these disagreements underscore that IDDSI and BET evaluate distinct but complementary aspects of food texture. IDDSI provides a practical, qualitative assessment of flow and handling properties relevant to swallowing safety, while BET offers a quantitative analysis of mechanical behavior under controlled deformation. Consequently, partial mismatches between both methods are not only expected but also reveal the complexity of texture, where structural, compositional, and rheological factors interact to influence the sensory and functional properties of food.

Although the study standardized measurement conditions, it must be kept in mind that temperature can notably impact textural behavior. Products containing fats or gelled components may appear firmer at lower temperatures (e.g., 5 °C), fitting with IDDSI level 4, but soften at higher temperatures (e.g., 40 °C), leading to BET profiles more consistent with level 3 [12,14].

A limitation inherent to both BET methods is their ability to predict the classification of only a subset of levels, rendering the predictions incomplete. These limitations underscore the need for more objective and reproducible instrumental methods to classify foods for people with OD. Early attempts using the Bostwick consistometer to correlate flow distance with apparent viscosity showed poor agreement when tested on three pureed baby foods [24]. More recent approaches have improved texture characterization, including ball back-extrusion, syringe flow, and fork drip tests. These methods have revealed two distinct objective ranges for IDDSI level 3, depending on the methodology used for levels classification [25]. Rheological analyses have also shown positive correlations between viscosity and syringe flow results for IDDSI levels 1–3 [6], supporting the ongoing refinement of IDDSI level descriptions.

**Figure 2:**
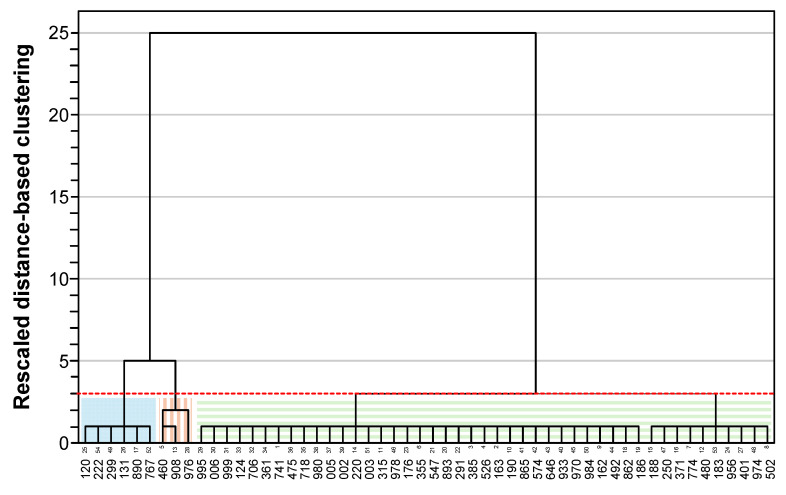
Clustering classification of the 54 commercial TMFs, as determined by the BET1 method (*X*-axis: samples coded with 3 digits). The dotted red line indicates the “cut distance” for determining the optimal number of clusters. The colored rectangles identify the clusters.

**Figure 3:**
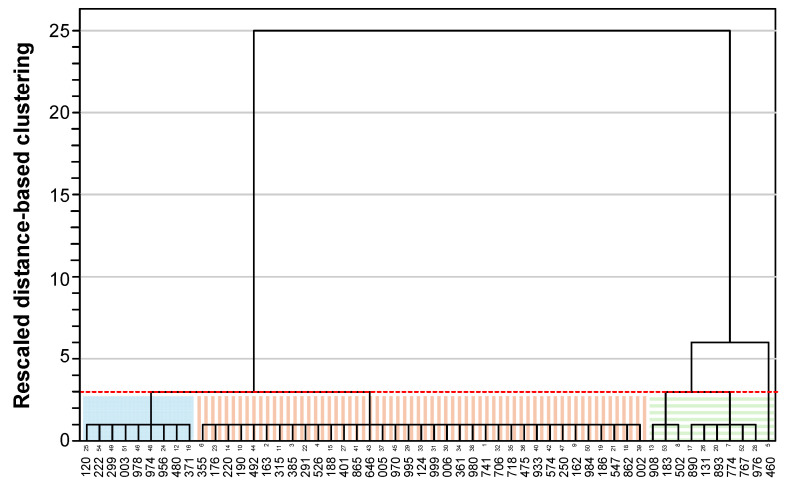
Clustering classification of the 54 commercial TMFs, as determined by the BET2 method (*X*-axis: samples coded with 3 digits). The dotted red line indicates the “cut distance” for determining the optimal number of clusters. The colored rectangles identify the clusters.

Building on these observations, a hierarchical cluster analysis was applied to the commercial food samples using the BET1 and BET2 methods grouping the samples accordingly. When four or five clusters were generated, one of them contained only a single sample. In contrast, when three clusters were created, the groups showed more balanced sample sizes. Figure 2 shows the three clusters generated using the BET1 method. The first cluster comprised 45 samples with firmness values ranging from 0.4 to 4.5 N, including all samples from IDDSI levels 0–2, as well as 19 samples from level 3 and 14 from level 4. The second cluster grouped 6 samples with firmness values between 5.2 and 7.4 N (4 from IDDSI level 4 and 2 from level 3), while the third cluster contained 3 samples with firmness 8.9–14.0 N, all corresponding to IDDSI level 4). Figure 3 depicts the three clusters identified by the BET2 method. The first cluster comprised 35 samples with consistency values ranging from 2.0 to 11.9 N × s, including all samples from IDDSI level 0–2, 15 from level 3 and 8 from level 4. The second cluster grouped 9 samples with consistency values between 13.2 and 24.0 N × s (6 from IDDSI level 3 and 3 from level 4), and the third cluster with 10 samples with consistency values ranging from 31.6 to 59.3 N × s, all belonging to IDDSI level 4.

The differentiation observed between the samples included in the clusters obtained by the BET1 (firmness) and BET2 (consistency) methods highlights the distinct mechanical and rheological behaviors that characterize TMFs designed for dysphagic people. The clusters derived from BET1 were mainly determined by the maximum force required to deform the sample, representing its firmness. This parameter reflects the instantaneous resistance to deformation and depends on the microstructural integrity of the food matrix, which is influenced by the degree of thickening, the type of hydrocolloid, protein or starch content, and the capacity of the system to bind and immobilize water [26]. Accordingly, the distribution of samples across the BET1 clusters reflects differences in composition and structural organization arising from the use of distinct ingredients and processing conditions. Cluster 1, which included most of the commercial samples (e.g., dairy drinks, juices, gelled waters, purées, and soft creams), was characterized by low firmness values. These products shared a high moisture content and a relatively simple or weak gel matrix, primarily stabilized by soluble carbohydrates, milk proteins, and hydrocolloids at low concentrations. Such microstructures promote high deformability and low mechanical resistance, consistent with the behavior described for weakly cross-linked gels and emulsions [27]. The prevalence of IDDSI levels 0–3 within this cluster supports this interpretation, as these levels correspond to thin to moderately thick textures that require minimal oral effort during swallowing. Cluster 2 grouped samples such as protein jelly, apple flakes, and chicken–carrot purée, characterized by intermediate firmness. These foods have a higher solid content and often include structural proteins (gelatin, caseinates) and starch, which form more stable networks through protein–polysaccharide interactions and partial gelation. This results in greater structural rigidity and lower deformation under stress [28]. Cluster 3 comprised the most rigid matrices, including items such as egg and broccoli purée, chicken with spinach, multigrain muesli, and fish gratin (high firmness). These products are rich in denatured proteins, starch granules, and lipid emulsions, components that interact to form dense, cohesive, and viscoelastic matrices with limited water mobility [29].

The BET2 method, based on consistency (integrated work during compression), provided a complementary approach that accounts for flow and deformation energy. The first BET2 cluster (low consistency) included similar products to BET1 Cluster 1: gelled waters, juices, shakes, and soft purées. These items combine high water activity and moderate viscosity but show limited viscoelastic recovery. Their rheological profile corresponds to shear-thinning fluids with low yield stress, which ensures easy flow but minimal structural retention [30]. The second cluster (intermediate consistency) contained more heterogeneous matrices, including protein jellies, thickened creams, gelled desserts, muesli, and vegetable-based purées. These foods typically have higher dry matter and hydrocolloid content, producing intermediate consistency. The incorporation of polysaccharides (e.g., xanthan, guar, modified starch) and milk proteins promotes elastic network formation, enhancing cohesiveness and resistance to flow [13]. Finally, the third cluster comprised the densest and most cohesive products, such as lentil purée powder, chicken with spinach, custard, honey-type juices, and natural yogurt (high consistency). These formulations exhibit a balance between water retention and network rigidity, characteristic of yield-stress fluids with strong viscoelastic and plastic components. Their composition—rich in proteins, starch, and lipids—supports the formation of stable semi-solid systems suitable for IDDSI level 4 (pureed or extremely thick foods).

The relatively high within-cluster heterogeneity observed in BET1 requires further consideration. This heterogeneity can be explained by several methodological and matrix-related factors. First, BET1 relies only on two basic parameters (firmness and adhesiveness) and on a specific probe geometry, which reduces the dimensionality of the information and limits its ability to capture subtle rheological differences in complex commercial formulations. According to work of Kaewsorn et al. [31], the precision and sensitivity of BET are highly dependent of probe geometry and test conditions, which may lead to non-discriminative values when applied to weakly structured or liquid matrices. Moreover, commercial formulations of OD-oriented foods vary widely in composition (texturizer agents, proteins, dietary fiber, and particulate matter), which differently affect viscosity, viscoelasticity, and bolus-like behavior; such compositional variability increases intra-cluster dispersion when only two instrumental parameters are considered [4]. Finally, weakly structured liquids show low mechanical contrast in compression/extrusion tests, reducing the signal-to-noise ratio of BET1 and favoring clustering together [32].

Overall, these clustering findings indicate that instrumental texture measurements are more effective at differentiating firmer, cohesive TMFs (levels 3–4) than fluid or mildly thickened foods (levels 0–2), and they underscore the value of combining multiple BET parameters for objective assessment of texture-modified foods. This may be due in part to the limited applicability of the BET1 method in liquids with small particle size or very fluid matrices, where the lack of internal structure likely leads to minimal mechanical resistance and, consequently, low and poorly discriminative values for firmness and adhesiveness. Similar challenges have been reported in rheological and tribological assessments of dysphagia thickeners, where the dominance of the liquid phase in weakly structured systems reduces the sensitivity of instrumental measurements [32].

Based on the clustering patterns and statistical outcomes, an alternative classification system for beverages and semi-solid foods is proposed, comprising three primary categories. Level 1 (Low Consistency) includes food with minimal structural resistance and low values for firmness, adhesiveness, and consistency. The overlapping texture parameters in these levels support their grouping. Level 2 (Intermediate Consistency) encompasses foods transitioning from thickened liquids to soft solids, with moderate firmness and the ability to maintain shape without excessive deformation. Level 3 (High Consistency Liquids and Semi-Solids) comprises foods with the highest firmness, cohesiveness, and cohesion work, providing structural integrity while remaining safe to swallow. Operational criteria for this three-class system are defined using quantitative cutoffs based directly on BET1 firmness and BET2 consistency. According to the cluster analysis, samples with BET1 firmness < 5.2 N and BET2 consistency < 12 N × s are classified as low consistency, generally corresponding to IDDSI levels 0–2, as well as some level 3 products with high water content and no added texturing agent. Samples with BET1 firmness between 5.5 and 7.4 N and BET2 consistency between 13 and 24 N × s are classified as medium consistency, generally including IDDSI level 3 products. Finally, samples exceeding **8**.9 N in BET1 firmness or 32 N × s in BET2 consistency are classified as high consistency, mainly corresponding to IDDSI level 4 and comprising dense, and cohesive matrices. This three-level system is consistent with the National Dysphagia Diet (NDD) framework, which proposes 4 viscosity-based levels [33]. These findings are also in line with those of Poursani & Razavi [9], who classified thickened fluids (IDDSI levels 0 to 4) using a combination of rheological and tribological parameters. Their cluster analysis revealed four distinct groups, suggesting that the traditional IDDSI levels may not fully capture the textural diversity of thickened liquids. This reinforces the idea that current classifications might benefit from refinement to better reflect the range of physical properties observed in commercial products. Similarly, Hadde et al. [25] established objective ranges correlating IDDSI levels 0 to 4 with apparent stress values obtained through ball back-extrusion testing, alongside syringe flow and fork drip tests. They identified two distinct ranges for IDDSI level 3, highlighting the potential need to refine IDDSI level descriptions. In another study, Zargaraan et al. [34] rheologically characterized 39 commercial food samples, including beverages, main courses, and desserts, and grouped them into five clusters based on rheological properties. Although IDDSI levels were not directly assessed, the nature of the samples suggests that they likely spanned IDDSI levels 0–4, providing practical insight into the range of textures present in commercial products.

Collectively, these findings underscore the challenges of objectively classifying TMFs for people with OD, particularly at intermediate and lower IDDSI levels. Further studies incorporating additional rheological and textural parameters, multi-dimensional clustering, and sensory evaluation are warranted to improve the grouping and discrimination of foods intended for these people.

## 4. Conclusions

This study assessed the effectiveness of instrumental back-extrusion tests (BET) for classifying commercial texture-modified foods (TMFs) for people with oropharyngeal dysphagia (OD) according to the IDDSI framework. Although BET alone did not fully achieve reliable and reproducible classification across all levels, the results indicate its potential as a valuable tool for evaluating TMFs. Foods assigned to IDDSI levels 0–3 were classified with reasonable accuracy, while level 4 products failed, likely due to complex composition and structural characteristics. These findings underscore the need to complement BET with additional instrumental approaches, such as rheological and tribological analyses, to achieve a more comprehensive understanding of TMF mechanical properties.

Hierarchical cluster analysis enabled the development of a simplified three-level classification system that aligns with the NDD framework: low consistency (IDDSI 0-2 and some level 3), intermediate consistency (IDDSI 3), and high consistency (IDDSI 4). Based on the findings of this study, a BET approach integrating four parameters—firmness, adhesiveness, cohesiveness, and cohesion work—is required to achieve accurate and reliable textural classification of commercial foods designed for individuals with OD.

Combining multiple instrumental parameters will enhance objectivity and reproducibility, highlighting the potential of integrating BET with complementary methods, including rheology, and tribology. Overall, BET represents a promising approach for the food industry, offering an objective, reproducible means to support the classification and development of TMFs while contributing to safer and more effective dietary management for patients with OD.

## Figures and Tables

**Table 1:** Data ranges for the textural parameters assessed using BET1 and BET2 in samples of commercial TMFs, classified according to their respective IDDSI levels (sample size in parentheses).

IDDSI Level	BET1		BET2			
	Firmness 1 (N)	Adhesiveness (N)	Firmness 2 (N)	Consistency (N × s)	Cohesiveness (N)	Cohesion Work (N × s)
0 (*n* = 6)	0.40 to 0.43	−0.21 to −0.23	0.11 to 0.21	1.99 to 2.62	−0.08 to −0.13	−0.01 to −0.03
1 (*n* = 4)	0.44 to 0.48	−0.22 to −0.25	0.14 to 0.18	2.13 to 2.66	−0.02 to −0.15	−0.02 to −0.05
2 (*n* = 2)	0.46 to 0.53	−0.25 to −0.27	0.24 to 0.31	4.19 to 5.44	−0.10 to −0.23	−0.20 to −0.31
3 (*n* = 21)	0.64 to 5.60	−0.27 to −4.43	0.15 to 1.03	2.72 to 17.98	−0.13 to −1.82	−0.08 to −2.62
4 (*n* = 21)	0.81 to 14.43	−0.31 to −12.91	0.33 to 6.85	5.71 to 69.76	−0.24 to −4.94	−0.34 to −6.84

**Table 2:** Texture properties of commercial TMFs according to the BET methods obtained across study groups categorized by IDDSI level (sample size in parentheses). Data are presented as mean values along with their corresponding standard deviations (*n* = 3).

	BET1		BET2			
IDDSI Level	Firmness 1 (N)	Adhesiveness (N)	Firmness 2 (N)	Consistency (N × s)	Cohesiveness (N)	Cohesion Work (N × s)
0 (*n* = 6)	0.41 ± 0.01 ^d^	−0.22 ± 0.00 ^a^	0.13 ± 0.03 ^d^	2.18 ± 0.23 ^d^	−0.09 ± 0.02 ^a^	−0.02 ± 0.01 ^a^
1 (*n* = 4)	0.46 ± 0.01 ^c^	−0.25 ± 0.00 ^b^	0.16 ± 0.01 ^d^	2.69 ± 0.32 ^cd^	−0.12 ± 0.02 ^b^	−0.02 ± 0.02 ^a^
2 (*n* = 2)	0.49 ± 0.03 ^c^	−0.26 ± 0.01 ^c^	0.22 ± 0.09 ^c^	3.75 ± 1.65 ^c^	−0.16± 0.07 ^b^	−0.16 ± 0.10 ^a^
3 (*n* = 21)	1.77 ± 1.52 ^b^	−1.14 ± 1.19 ^d^	0.50 ± 0.27 ^b^	8.74 ± 4.56 ^b^	−0.62 ± 0.42 ^c^	−0.93 ± 0.60 ^b^
4 (*n* = 21)	3.99 ± 3.75 ^a^	−2.67 ± 3.26 ^e^	1.67 ± 1.33 ^a^	24.61 ± 15.49 ^a^	−1.71 ± 1.29 ^d^	−2.26 ± 1.77 ^c^

Different superscripts in the same column indicate statistically different means by Dunnett’s C test (*p* < 0.05).

**Table 3:** Discriminant analysis classification of TMF samples by the BET1 method across IDDSI levels (values expressed as %). Shaded cells indicate the highest classification percentage in each observed IDDSI level.

		Predicted IDDSI Level				
		0	1	2	3	4
Observed IDDSI level	0	100	0.0	0.0	0.0	0.0
	1	0.0	100	0.0	0.0	0.0
	2	0.0	0.0	50.0	50.0	0.0
	3	0.0	0.0	0.0	86.4	13.6
	4	0.0	0.0	0.0	63.6	36.4

**Table 4:** Discriminant analysis classification of TMF samples by the BET2 method across IDDSI levels (values expressed as %). Shaded cells indicate the highest classification percentage in each observed IDDSI level.

		Predicted IDDSI Level				
		0	1	2	3	4
Observed IDDSI level	0	100.0	0.0	0.0	0.0	0.0
	1	0.0	100.0	0.0	0.0	0.0
	2	0.0	0.0	50.0	50.0	0.0
	3	0.0	0.0	0.0	95.5	4.5
	4	0.0	0.0	0.0	45.5	54.5

## Data Availability

The original contributions presented in this study are included in the article/Supplementary Materials. Further inquiries can be directed to the corresponding author.

## Supplementary Materials

The following supporting information can be downloaded at: https://www.mdpi.com/article/10.3390/foods14213741/s1, Table S1: Type and description of foodstuffs, composition (as a ready-to-eat product), measurement temperature, IDDSI level and results of back-extrusion test (BET) for selected TMF samples suitable for oropharyngeal dysphagia; Figure S1: Discriminant analysis plot for commercial TMFs using the BET1 method, showing groups according to IDDSI levels; Figure S2: Discriminant analysis plot for commercial TMFs using the BET2 method, showing groups according to IDDSI levels.

## Abbreviations

The following abbreviations are used in this manuscript:

OD	Oropharyngeal Dysphagia
TMFs	Texture-Modified Foods
BET	Back-Extrusion Test
IDDSI	International Dysphagia Diet Standardization Initiative
NDD	National Dysphagia Diet Task Force
