# Supplementary material for: Textural Classification of Commercial Foodstuffs for Dysphagia Using Back-Extrusion Test
Source: Foods. 2025 Oct 31;14(21):3741. doi: 10.3390/foods14213741 (PMC12611084; doi:10.3390/foods14213741)
Supplement: Supplementary file 1 [file foods-14-03741-s001.zip › foods-3891587-supplementary.pdf]

Supplementary materials

Table S1. Type and description of foodstuffs, composition (as a ready-to-eat product), measurement temperature, IDDSI level and results of back-extrusion test (BET) for selected TMF samples suitable for oropharyngeal dysphagia**.**

			Composition*						BET1		BET2			
**Sample number**	**Type**	**Description (brand)**	**proteins** (%)	**fats** (%)	**carbohy drates** (%)	**dietary fiber** (%)	**Measure T** (°C)	**IDDSI level**	**Firmness1** (N)	**Adhesiveness** (N)	**Firmness2** (N)	**Consistency** (N x s)	**Cohesiveness** (N)	**Cohesion work** (N x s)
1	Beverage	Dairy drink, strawberry flavor (Danacol®)	2.70	1.10	4.30	—	5	1	0.45±0.01	-0.24±0.01	0.15±0.01	2.63±0.11	-0.12±0.01	-0.00±0.03
2	Jelly	Gelled water, lemon flavor (Vegenat®)	0.00	0.00	1.00	1.90	5	4	1.10±0.01	-0.38±0.01	0.69±0.01	11.97±0.21	-0.60±0.01	-0.82±0.03
3	Jelly	Gelled water, orange flavor (Vegenat®)	0.00	0.00	2.10	1.50	5	4	1.07±0.01	-0.37±0.01	0.56±0.01	9.82±0.11	-0.30±0.01	-0.73±0.02
4	Jelly	Gelled water, lemon & apricot flavor (Vegenat®)	0.00	0.20	1.50	1.80	5	4	1.06±0.01	-0.35±0.01	0.58±0.00	10.04±0.05	-0.50±0.00	-0.75±0.03
5	Jelly	Protein jelly, strawberry flavor (HACENDADO)	6.00	0.00	3.80	—	5	4	12.18±0.50	-5.19±1.60	6.14±0.62	56.44±8.24	-1.57±0.94	-1.80±0.83
6	Purée	Apple and plum purée (Campofrío®)	0.40	0.00	14.00	3.20	5	3	0.96±045	-0.44±019	0.45±0.21	7.89±3.81	-0.34±0.02	-0.96±0.20
7	Cream	Hyperproteic cream mix and vanilla (T.Aliment)	8.59	1.85	6.57	1.29	5	4	2.77±0.04	-1.60±0.04	2.65±0.01	41.73±0.18	-2.86±0.50	-4.10±0.02
8	Jelly	Gelled water, raspberry flavor (Campofrío®)	0.00	0.00	0.80	0.80	20	4	4.20±0.02	-1.07±0.02	2.43±0.11	31.51±1.14	-2.18±0.06	-1.33±0.24
9	Purée	Apple and banana purée (Campofrío®)	0.30	0.20	16.00	1.70	20	3	0.78±0.012	-0.31±0.02	0.42±0.24	7.10±3.08	-0.35±0.00	-0.60±0.16
10	Purée	Apple and pear purée (Campofrío®)	0.50	0.00	15.00	2.20	20	3	1.07±0.01	-0.47±0.01	0.51±0.06	9.02±1.15	-0.50±0.11	-0.82±0.19
11	Purée	Fruit salad purée (Campofrío®)	0.50	0.00	15.00	2.10	20	3	1.20±0.01	-0.58±0.01	0.66±0.03	11.61±0.54	-0.70±0.04	-1.09±0.06
12	Purée	Banana and cereal purée (MITATA)	0.30	0.30	16.00	2.20	20	4	2.83±0.03	-1.82±0.01	1.37±0.03	22.16±0.52	-1.36±0.03	-1.82±0.05
13	Purée	Apple flakes (Vegenat®)	0.40	0.16	18.20	2.40	20	4	8.87±0.40	-9.08±0.28	2.95±0.12	43.91±0.69	-4.67±0.06	-6.31±0.08
14	Purée	Fruit, milk and cereal mix, cereal & banana flavor (Campofrío®)	3.10	2.30	15.00	1.10	40	4	1.61±0.16	-0.79±0.22	0.43±0.04	7.62±1.38	-0.42±0.16	-0.69±0.38
15	Purée	Fruit, milk and cereal mix, coffee & biscuit flavor (Campofrío®)	2.90	2.20	13.00	1.00	40	4	1.99±0.04	-1.16±0.05	0.59±0.01	10.49±0.14	-0.70±0.01	-1.07±0.02
16	Purée	Lentil purée powder (Vegenat®)	6.90	2.98	7.90	1.52	40	4	2.37±0.05	-1.35±0.06	1.46±0.01	24.10±0.21	-1.72±0.02	-2.37±0.03
17	Purée	Eight cereals with muesli (T.Aliment)	4.00	1.70	14.40	0.60	40	4	7.43±0.11	-5.94±0.08	2.06±0.03	33.98±0.58	-3.48±0.03	-4.67±0.02
18	Cream	Vegetable cream 1 (MITATA)	0.80	1.20	8.20	1.50	40	3	0.74±0.025	-0.28±0.24	0.35±0.14	5.81±2.52	-0.21±0.01	-0.32±0.03
19	Cream	Asparagus cream, powder (Vegenat®)	2.88	1.76	12.50	1.40	40	4	0.89±0.03	-0.32±0.01	0.35±0.02	5.99±0.32	-0.23±0.01	-0.35±0.03
20	Cream	Vegetable cream 2, powder (Vegenat®)	2.96	1.74	11.78	1.88	40	4	1.17±0.03	-0.39±0.01	2.16±0.01	35.95±0.22	-3.64±0.01	-5.26±0.02
21	Minced dish	Chicken in *chilindrón* sauce (MITATA)	3.60	2.00	6.80	2.00	40	3	0.97±0.01	-0.42±0.01	0.34±0.02	6.08±0.94	-0.39±0.04	-0.61±0.19
22	Minced dish	Chickpea stew (MITATA)	4.10	2.70	7.30	3.50	40	4	1.18±0.03	-0.42±0.02	0.61±0.02	9.82±0.36	-0.53±0.02	-0.82±0.04
23	Minced dish	Rice and seafood (MITATA)	3.00	2.40	8.20	1.00	40	3	1.35±0.02	-0.79±0.02	0.47±0.01	8.40±0.30	-0.67±0.03	-1.02±0.02
24	Minced dish	Turkey, *courgette* and potato purée (Campofrío®)	6.50	4.50	11.00	0.80	40	3	3.67±0.11	-3.05±0.08	0.72±0.05	12.81±0.87	-1.19±0.07	-1.77±0.08
25	Minced dish	Chicken with spinach (T.Aliment)	6.68	2.76	8.40	0.84	40	3	5.23±0.16	-4.13±0.14	0.91±0.06	15.97±0.92	-1.04±0.17	-1.51±0.20

Sample number	Type	Description (brand)	Composition*				Measure T (°C)	IDDSI level	BET1		BET2			
			proteins (%)	fats (%)	carbohy drates (%)	dietary fiber (%)			Firmness1 (N)	Adhesiveness (N)	Firmness2 (N)	Consistency (N x s)	Cohesiveness (N)	Cohesion work (N x s)
26	Minced dish	Egg and broccoli (T.Aliment)	5.80	2.40	6.60	1.50	40	4	5.57±0.05	-4.45±0.12	2.10±0.03	34.40±0.64	-2.81±0.04	-3.96±0.04
27	Minced dish	Beef, vegetables and rice purée (Campofrío®)	6.50	4.00	11.00	<0.5	40	3	3.53±0.09	-3.03±0.13	0.58±0.06	10.57±0.74	-1.10±0.08	-1.68±0.09
28	Minced dish	Chicken and carrot purée, low sodium (Cyranie nutrition)	2.40	1.12	13.40	0.00	40	4	14.00±0.41	-12.70±0.42	2.39±0.01	36.62±0.20	-2.92±0.02	-3.99±0.05
29	Beverage	95% milk-cocoa shake (EROSKI)	3.20	1.20	9.30	0.60	5	0	0.40±0.00	-0.22±0.00	0.12±0.03	2.02±0.00	-0.08±0.00	-0.56±0.19
30	Beverage	Orange juice (HACENDADO)	0.70	0.10	9.90	0.60	5	0	0.40±0.00	-0.22±0.00	0.11±0.00	2.13±0.01	-0.08±0.00	-0.01±0.00
31	Beverage	Protein coffee shake (alpro®)	6.00	2.80	5.10	0.90	5	0	0.41±0.00	-0.22±0.00	0.11±0.01	1.99±0.16	-0.08±0.01	-0.01±0.00
32	Beverage	Complete food with fiber, vanilla flavor (FontActiv®)	23.00	23.00	40.00	7.00	5	0	0.41±0.00	-0.23±0.00	0.19±0.01	2.62±0.01	-0.13±0.00	-0.03±0.00
33	Beverage	90% milk shake, strawberry flavor (EROSKI)	2.90	0.50	10.00	0.00	5	0	0.41±0.00	-0.22±0.00	0.11±0.02	2.02±0.08	-0.08±0.01	-0.01±0.00
34	Beverage	Protein shake, strawberry flavor (HACENDADO)	7.10	0.40	4.80	—	5	0	0.43±0.00	-0.22±0.00	0.12±0.01	2.18±0.03	-0.09±0.00	-0.02±0.00
35	Beverage	Complete high-protein, high-calorie diet, apricot & peach flavor (FRESENIUS KABI)	7.50	4.70	19.50	0.10	20	1	0.45±0.00	-0.25±0.00	0.14±0.00	2.41±0.05	-0.12±0.00	-0.02±0.00
36	Beverage	Complete polymeric hyperproteic, hypercaloric formula, without fiber, coffee flavor (FontActiv®)	10.00	7.50	23.00	—	5	1	0.45±0.01	-0.24±0.00	0.16±0.01	2.66±0.08	0.00±0.02	0.00±0.01
37	Beverage	Tomato juice (HACENDADO)	0.90	0.00	3.30	0.40	5	1	0.40±0.00	-0.22±0.00	0.11±0.00	2.13±0.10	-0.08±0.01	-0.01±0.00
38	Beverage	High-calorie and high-protein diet drink (Nestlé)	10.00	7.00	24.30	—	5	2	0.46±0.00	-0.25±0.00	0.13±0.01	2.24±0.10	-0.10±0.01	-0.02±0.01
39	Beverage	Orange juice (HACENDADO) nectar 1 consistency with thickener (FontActiv®)	0.68	0.10	13.10	0.58	5	2	0.52±0.00	-0.26±0.00	0.30±0.01	5.16±0.04	-0.22±0.01	-0.29±0.00
40	Beverage	Orange juice (HACENDADO) nectar 2 consistency with thickener (FontActiv®)	0.72	0.10	10.73	1.16	5	3	0.64±0.01	-0.27±0.01	0.24±0.01	4.19±0.21	-0.19±0.01	-0.21±0.02
41	Beverage	Dense drink, vanilla flavor (Nestlé)	10.00	7.70	23.00	—	5	3	0.65±0.01	-0.33±0.00	0.18±0.01	3.27±0.10	-0.24±0.01	-0.36±0.02
42	Beverage	Dense drink, coffee flavor (Nestlé)	10.00	7.70	23.00	—	5	3	0.67±0.01	-0.34±0.00	0.22±0.01	4.00±0.13	-0.30±0.02	-0.47±0.04
43	Beverage	Dense drink for diabetes, vanilla flavor (Nestlé)	10.00	6.80	13.50	2.40	5	3	0.71±0.03	-0.33±0.01	0.18±0.01	3.12±0.14	-0.22±0.03	-0.32±0.06
44	Beverage	Tomato juice (HACENDADO) nectar 1 consistency with thickener (FontActiv®)	0.92	0.00	4.25	0.96	5	3	0.79±0.01	-0.30±0.01	0.47±0.06	8.27±1.15	-0.44±0.10	-0.72±0.19
45	Cream	Four vegetable cream (HACENDADO)	0.60	2.60	4.70	1.00	40	3	0.81±0.01	-0.32±0.00	0.17±0.01	3.00±0.19	-0.14±0.01	-0.10±0.02
46	Beverage	Tomato juice (HACENDADO) honey 1 consistency with thickener (FontActiv®)	0.94	0.00	5.16	1.50	5	3	1.32±0.09	-0.57±0.01	0.82±0.01	13.96±0.24	-0.85±0.01	-1.25±0.02
47	Beverage	Dense drink for diabetes, coffee flavor (Nestlé)	10.00	6.80	13.50	2.40	5	3	1.90±0.02	-1.44±0.02	0.25±0.01	4.56±0.31	-0.45±0.01	-0.76±0.03
48	Custard	Custard, vanilla flavor (DANONE)	3.10	2.90	17.70	—	5	3	4.21±0.22	-2.53±0.20	0.74±0.01	13.04±0.26	-0.94±0.04	-1.38±0.07

Sample number	Type	Description (brand)	Composition*				Measure T (°C)	IDDSI level	BET1		BET2			
			proteins (%)	fats (%)	carbohydrates (%)	dietary fiber (%)			Firmness1 (N)	Adhesiveness (N)	Firmness2 (N)	Consistency (N x s)	Cohesiveness (N)	Cohesion work (N x s)
49	Beverage	Complete high-calorie, high-protein diet with honey-type fiber, vanilla flavor (Nestlé)	12.50	12.00	22.00	—	5	3	5.31±0.09	-4.13±0.04	0.90±0.02	14.98±0.28	-1.54±0.04	-2.22±0.02
50	Cream	Homemade cream of 7 vegetables with Emmental cheese (Gallina Blanca)	2.00	2.00	4.00	1.90	40	4	0.82±0.33	-0.32±0.00	0.41±0.10	7.13±1.43	-0.35±0.02	-0.56±0.27
51	Beverage	Orange juice (HACENDADO) honey 1 consistency with thickener (FontActiv®)	0.68	0.10	14.60	0.57	5	4	0.94±0.01	-0.34±0.01	0.54±0.02	9.39±0.24	-0.51±0.02	-0.83±0.03
52	Minced dish	Fish gratin, low sodium (Cyranie nutrition)	2.80	1.04	13.60	0.00	40	4	2.37±0.01	-1.27±0.01	3.01±0.00	43.84±0.08	-3.19±0.00	-4.34±0.03
53	Jelly	Gelled water, orange flavor (Nestlé)	0.00	0.00	0.20	—	5	4	2.90±0.18	-1.05±0.08	2.87±0.25	38.28±5.28	-1.66±0.10	-1.65±0.11
54	Yoghurt	Plain yoghurt (DANONE)	3.30	3.30	4.30	—	5	4	7.79±0.05	-3.87±0.12	2.08±0.03	29.02±0.64	-1.92±0.04	-1.82±0.04

* Data from the product labels or the product reconstituted in accordance with the manufacturer's instructions.

Data represents the mean ± standard deviation (*n* = 3)

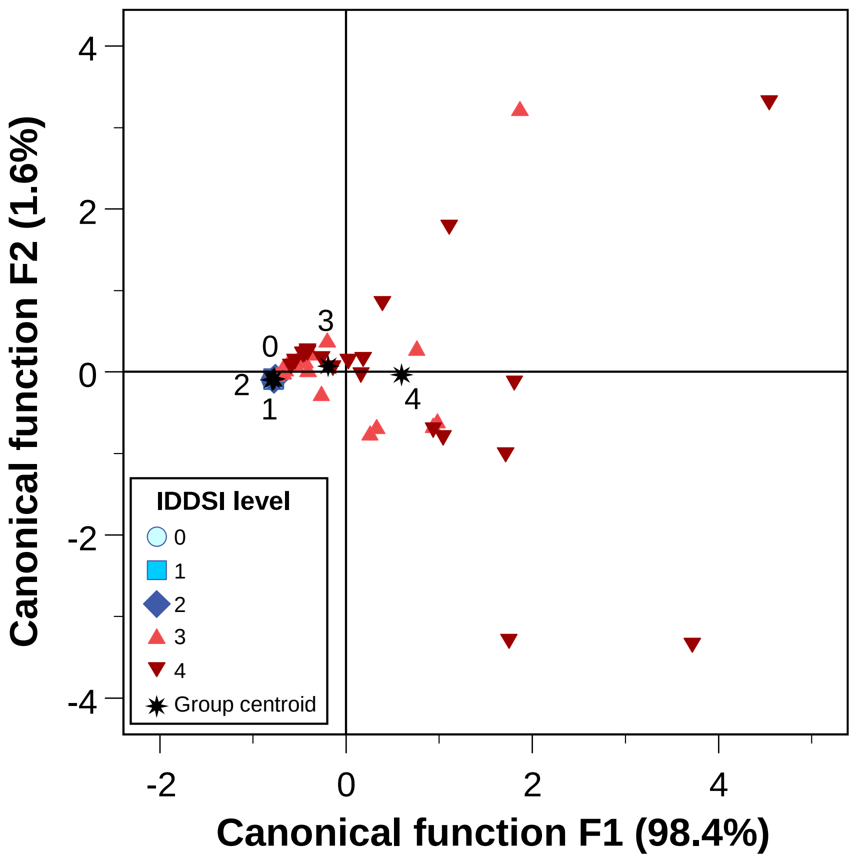

Figure S1. Discriminant analysis plot for commercial TMFs using the BET1 method, showing groups according to IDDSI levels.

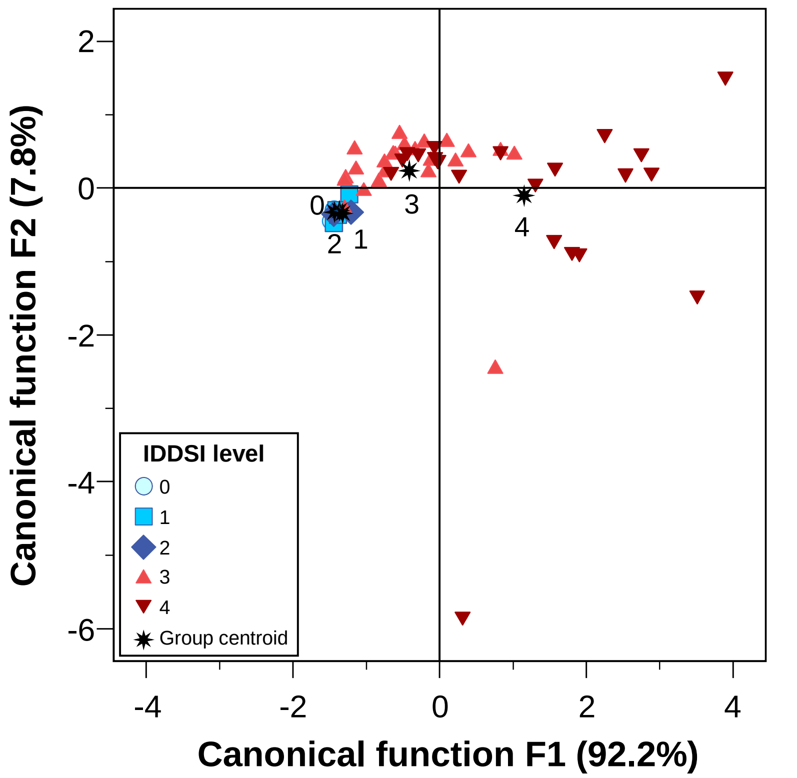

Figure S2. Discriminant analysis plot for commercial TMFs using the BET2 method, showing groups according to IDDSI levels.
